# Kinematics and muscle activity of the lower limb during single-leg stance on the two sides of the Togu Jumper

**DOI:** 10.3389/fphys.2023.1049035

**Published:** 2023-02-15

**Authors:** Petra Mayer, Balázs Sebesi, Kitty Vadász, József Laczkó, Norbert Zentai, Bence Balázs, Márk Váczi

**Affiliations:** ^1^ Doctoral School of Biology and Sportbiology, University of Pécs, Pécs, Hungary; ^2^ Institute of Sport Sciences and Physical Education, Faculty of Sciences, University of Pécs, Pécs, Hungary; ^3^ Institute of Mathematics and Informatics, Faculty of Sciences, University of Pécs, Pécs, Hungary

**Keywords:** instable surface, lower extremity, EMG, acceleration, balance

## Abstract

**Purpose:** Togu Jumper is a both sides utilized balance training device, which consists of an inflated rubber hemisphere attached to a rigid platform. It has been shown to be effective in improving postural control but there are no recommendations for the usage of the sides. Our aim was to examine leg muscle activity and kinematics in response to a single-leg stance on the two sides of the Togu Jumper and the floor.

**Methods:** In 14 female subjects, linear acceleration of leg segments, segmental angular sway, and myoelectric activity of 8 leg muscles were recorded in the three stance conditions.

**Results:** Except gluteus medius and gastrocnemius medialis, all muscles were more active when balancing on either Togu Jumper side compared to the floor (*p* < 0.001), but there was no difference between the two sides in any muscles. Linear acceleration was the greatest in the frontal plane on the flat Togu side in the case of the foot (*p* < 0.001). Pelvis acceleration was unaffected by the balance conditions. Segmental angular sway was the greatest in the frontal plane, on the bladder side in the foot segment (*p* < 0.001). No difference was found among the three conditions (all *p* > 0.05) in the case of the shank, thigh, and pelvis.

**Conclusion:** The use of the two Togu Jumper sides produced different balance strategies in the foot segment and induced no difference in equilibrium procedures at the level of the pelvis.

## Introduction

The use of unstable surfaces in proprioceptive training is a widely accepted modality to improve balance, and with the integrated activity of the surrounding muscles, to improve joint stability (Ferreira et al., 2011). Exercising on balance devices is an important part of the rehabilitation process (McKeon et al., 2008; Zech et al., 2009; Mattacola et al., 2002) and the prevention of injuries in both athletes and untrained individuals as well as in patients (Verhagen et al., 2004).

In recent years, several balance training devices have been developed such as soft mats, tilt boards, wobble boards, the both sides utilized Togu® Jumper® (TJ), and are used for the aforementioned purposes (Mattacola et al., 2002; Behrens et al., 2015; Fitzgerald et al., 2010). Manufacturers provided TJs with a flat, solid plastic base and an inflatable bladder, corresponding to a halved Swiss ball (www.togu.de). Due to its unique design, TJ can be used as a compliant surface on the bladder side and a combination of a wobble board and a compliant surface on the flat side. TJ was first introduced in 2008, and rapidly became a popular fitness and athletic training product.

Numerous studies reported the favorable effects of TJ exercises on postural control, balance, low back, or knee pain, and muscle strength. Despite the excessive data on the long-term effectiveness of TJ exercises, however, previous literature failed to extensively study whether the effects are surface-specific (Yaggie and Campbell 2006; Romero-Franco et al., 2012; Martinez-Amat et al., 2013; Seo and Park 2014; Codorean et al., 2016; Freyler et al., 2016; Kowalczyk et al., 2019). TJ is commonly used in proprioceptive and balance training targeting the knee (Codorean et al., 2016; Ju et al., 2015; Myer et al., 2008), ankle (Clug et al., 2016; Ju and Park 2017), and core stability (Ruiz et al., 2005; Seo and Park., 2014; Ipekoglu et al., 2018) but there are no recommendations for the usage of a particular side of the TJ in special therapy or exercise training. Hof (2007) named three different theoretical mechanisms which allow for balance maintenance during standing tasks. The first mechanism is to relocate the center of pressure within the base of support which is described by the inverted pendulum model. If the base of support is too small for the first mechanism, the second mechanism comes into force according to which, a counter-rotation mechanism can be applied for the maintaining of balance, which extends the area in which balance can be regained by up to 6 cm beyond the base of support. This counter-rotation mechanism is accomplished by movements of free segments around the center of mass, i.e., arms, upper trunk and/or the free leg. The third mechanism was to apply an external force by leaning to something which is not relevant in the case of our investigation. Furthermore, exercising on a flat versus a bladder surface may induce different balancing strategies to maintain posture, and the kinematic and dynamic mechanisms of these strategies are unclear. Gruber et al., (2006) observed that, with a fixed ankle joint, the control of balance shifts from the shank to the thigh muscles. Standing on the flat surface of the TJ may provoke similar balancing strategies (i.e., induce more angular movements in the knee or in upper segments), except that the bladder surface of the TJ that contacts the floor can also rotate easily. In addition, the center of this rotation can move in the horizontal plane. In contrast, standing on the bladder side, greater angular motions can be provoked in the ankle joint. Moreover, because the bladder side is also very compliant, the intrinsic foot joints (the subtalar joint, midtarsal or Chopart’s joint, Lisfranc joint) and their supporting muscles may also play an important role to maintain balance. The varying topography and congruency between articular surfaces of the foot joints result in a continuously changing center of rotation during motion (Harrold and Abboud 2018). These conditions and the unique material and surface of the two sides of the TJ could lead to different progression of the balancing process on one leg.

The closest to the focus of the present work is the study by Laudner and Koschnitzky (2010), in which they measured the electrical activity of the ankle muscles using a both sides utilized balance trainer, with the flat side either resting on the floor or facing up, and found no significant difference between the two conditions. To our knowledge, there have been no studies varying the two sides of the TJ and simultaneously monitoring leg muscle activation and kinematics around the ankle, knee, and hip joint. Understanding the neurokinematical strategies of balancing on the bladder *versus* the flat surface of the TJ is important in order to target stability in a specific joint.

In the present investigation, we tested the hypothesis that the magnitudes of upper and lower leg muscle activations as well as segmental sways and accelerations are different among standing on the floor, on the flat side, and on the bladder side of the TJ during a single leg balancing test.

## Materials and methods

### Subjects

Because of the higher incidence of ankle sprain (Doherty et al., 2014) and ACL injuries (Moltavo et al., 2019) in females compared with males, fourteen healthy female physical education students (age: 22 years, range: 21–27 years; height: 167 cm, range: 148–179 cm; mass: 59 kg, range: 52–72 kg, years of training: 9.7 ± 2.6 years, track and field athletes, football and handball players not at national level) were involved in our study. The following inclusion criteria were used: physically active (minimum 2 hours per week), female, age between 18 and 30 years. The exclusion criteria were current injuries in the spine, hip, knee, ankle, previous surgeries in these joints, vestibular abnormalities, and participation in competitive sport. To determine *a-priori* sample size, we used the study by Cimadoro et al. (2013), who compared postural sway and EMG activity during balancing on different training devices. In this study, a one-way ANOVA (similar to our design) with four levels of surface variation as independent factor revealed that thirteen participants were enough to produce very large effect sizes (*d* > 1.47) in most, or a large effect size (*d* = 0.96) in one variable. Subjects gave their written informed consent according to the Declaration of Helsinki after receiving both a verbal and a written explanation of the experimental protocol and its potential risks. The University Ethics Committee approved the protocol (approval number: 7961-PTE2019).

### Experimental protocol and data processing

**FIGURE 1:**
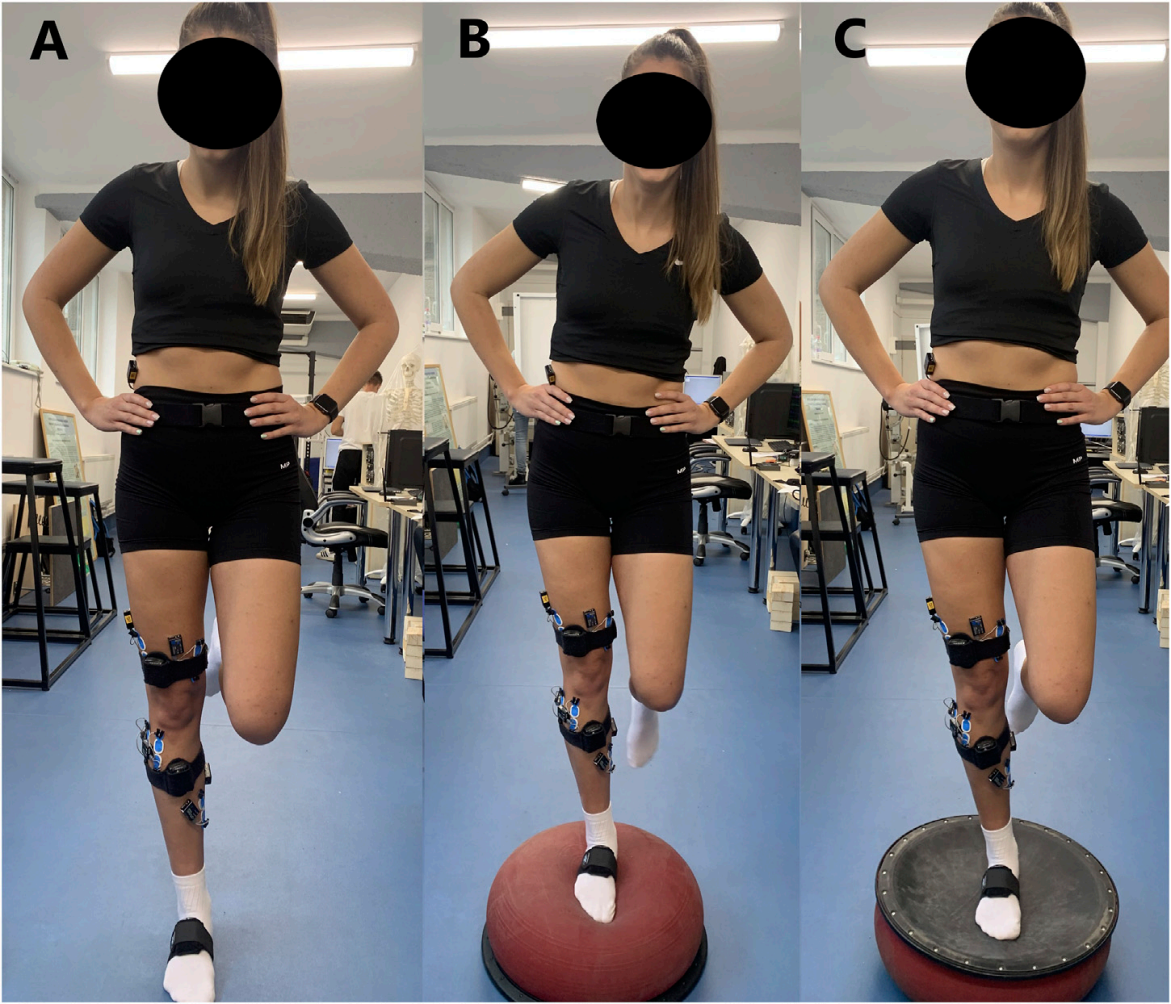
Shows a subject performing single legged balance test on different surfaces. **(A)** Floor, **(B)** Bladder side of the Togu Jumper, **(C)** Flat side of the Togu Jumper.

Subjects were asked to perform unipedal quiet stance trials on their dominant leg on three different surfaces: the floor, the bladder side of the TJ, and the flat side of a TJ. Initially, anthropometric data were collected followed by the setting up of EMG electrodes and motion tracking sensors on the appropriate leg parts. At first, each participant completed the trials on the floor, then randomly performed the tests on the two sides of the TJ. The order of the trials in the bladder and the flat conditions were alternated and the condition of the first trial was counterbalanced. For all trials, subjects were barefoot with their eyes open focusing on a target 2 m in front of them with hands-on their hips and the opposite leg flexed under the body without touching the balancing leg, which was allowed to be slightly flexed (Figure 1). If at any point the subject broke from this position (e.g., the subject’s contralateral leg touched the ground), the data were discarded and the trial was recommenced. Each trial lasted 10 s and was repeated three times with a 1-min break between the trials (Laudner and Koschnitzky 2010). During the rest periods, participants were required to sit down on a chair and stay calm.

### Surface electromyography (EMG)

During the balancing trials, EMG data were collected telemetrically. The skin was carefully prepared by shaving and cleaning with alcohol. Dual Ag/AgCl surface electrodes (Noraxon, Scottsdale, United States of America) were positioned on eight lower extremity muscles, namely tibialis anterior (TA), peroneus longus (PL), soleus (SOL), gastrocnemius medialis (GastM), biceps femoris (BF), vastus lateralis (VL), vastus medialis (VM), and gluteus medius (GM). EMG electrode positioning on the muscles was carried out by the SENIAM recommendations (www.seniam.org). The input impedance for our EMG amplifyer was >100 Mohm. The common-mode rejection radio was >100 dB. Interelectrode distance was 20 mm. Electrodes were kept in their place throughout the balance tests. EMG signals were collected (Noraxon, Scottsdale, United States of America, sampling frequency: 2000 Hz), band pass filtered (20–500 Hz), and processed with the root mean square (RMS) technique, using 50-m moving window. For each muscle, the mean RMS EMG activity was thereafter calculated considering the entire 10-s-long trial.

### Kinematics

In the three conditions, balance performance was quantified using the segmental sway approach (Curtis et al., 2015; Ivusza et al., 2022): the foot, the shank, and the thigh of the stance leg as well as the pelvis was equipped with 3D motion tracking sensors (Noraxon United States. Inc., Scottsdale, United States). The sensors include three orthogonally mounted gyroscopes to sense 3D angular motions. Thus, we were able to determine angular deviation from the vertical axis (orientation angle) in the selected plane. The vertical axis was considered 0°, which was calibrated by requesting the participant to stand still on two legs in upright position for 10 s. We followed the manufacturer’s recommendations concerning sensor positioning and calibration. Briefly, sensors were affixed to the foot: upper foot, slightly below the ankle; for shank: on the tibia bone; for thigh: on the lower quadrant of quadriceps, slightly above the knee cap, and area of lowest muscle displacement in motion; pelvis: on the bony area of sacrum. During the tests, the segmental orientation angle and linear acceleration data were recorded with respect to time at 100 Hz sampling frequency in both the frontal and the sagittal plane, using MyoMOTION hardware (Noraxon United States. Inc., Scottsdale, United States).

Linear acceleration data were processed in two anatomical planes: in frontal plane, that divides the body into anterior and posterior portion and in sagittal plane that divides the body into right and left sections. In frontal plane, the medial-lateral, in sagittal plane, the anterior-posterior displacement of the sensors were detected (frontal = medial-lateral direction, sagittal = anterior-posterior direction) for the 10 s. Raw linear acceleration signals were smoothed, rectified, and low-pass Butterworth filtered at 50 Hz. The mean linear accelerations were calculated by the myoRESEARCH 3.18 software (Noraxon United States of America. Inc., Scottsdale, United States of America) for the entire 10-s-long test periods.

The length of the orientation angle—time curve was calculated off-line in the entire 10-s-long test period, using the Pythagorean theorem as follows:

$$\sum_{k}^{n}\sqrt{\left({\left[\alpha \left(k\right)-\alpha \left(k-1\right)\right]}^{2}+0.0001\right)}$$

where *α* = segmental orientation angle (°) with respect to the vertical axis (0°), and *k* represents the actual data point on the angle—time curve. The value of 0.0001 was obtained by squaring the 0.01-s sampling interval (at 100 Hz sampling frequency) on the horizontal axis of the angle—time curve. Using this data processing, shorter orientation angle—time curve demonstrated less segmental angular sway and better balance performance. A representative orientation angle—time curve is shown in a recent study published by our laboratory (Ivusza et al., 2022).

All kinematic and EMG data were synchronized and processed using myoRESEARCH 3.18 software (Noraxon United States. Inc., Scottsdale, United States). Averages of the three trials for each condition were used for further statistical analysis.

### Statistical analyses

Means and standard deviations were computed for the measured and calculated variables. All data were checked for normality (Shapiro-Wilk test). Variables that did not show normal distribution were log transferred to obtain normality. Relative EMG activities of the muscles across conditions were compared with two-factorial ANOVA, using muscle (TA, PL, SOL, GastM, VL, VM, BF, GM) and condition (floor, bladder, and flat) as independent variables. To test interactions among conditions (floor, bladder, and flat), and body segment (foot, shank, thigh, and pelvis) two-way ANOVAs were performed for linear acceleration and segmental angular sway, obtained in frontal and sagittal planes during the 10-s-long balance test. In any pairwise comparisons, the Bonferroni correction was used. The statistical significance was set at *p* = 0.05.

## Results

### EMG

**FIGURE 2:**
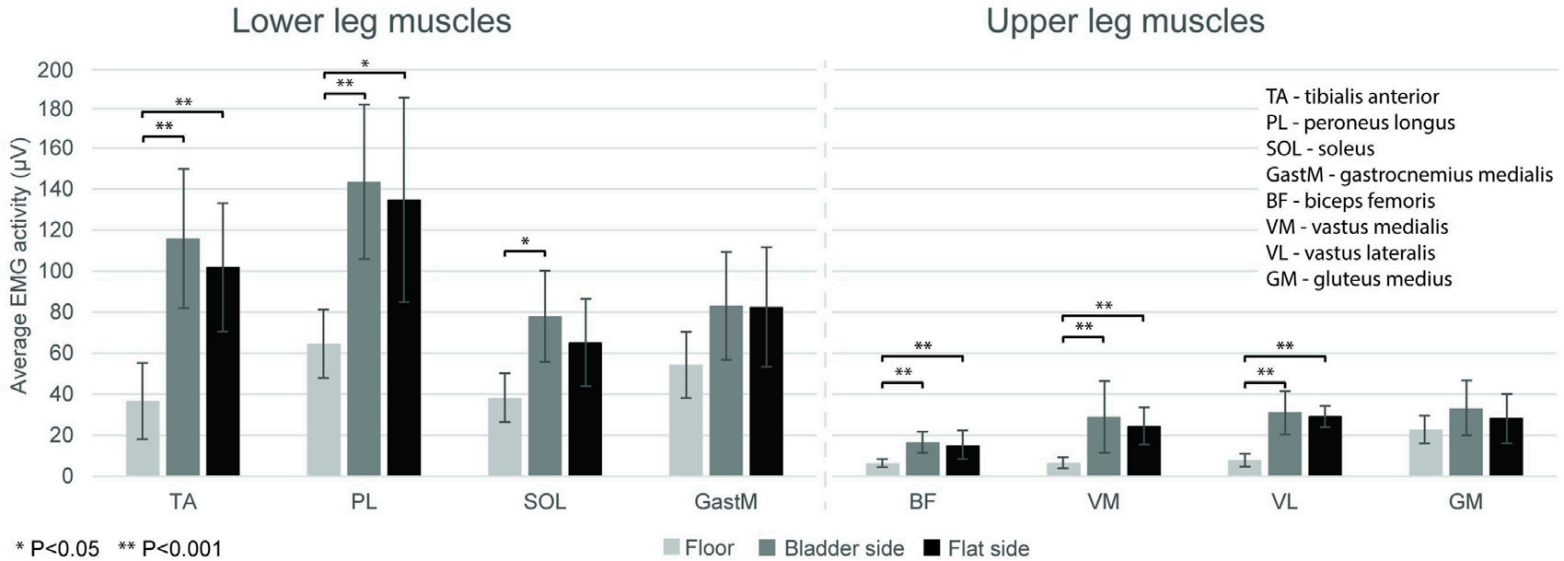
Average EMG activity (mean ± SD) in the three conditions (floor, and bladder and flat side of the Togu Jumper) for lower and upper leg muscles during the 10-s single leg balance trials. Horizontal bars indicate statistically significant differences *(*p* < 0.05) and ** (*p* < 0.001).

In EMG activity, there was significant main effect for surface (*F* = 123.69, *p* < 0.001) and muscle (*F* = 69.87, *p* < 0.001) with surface by muscle interaction (*F* = 13.18, *p* < 0.001). The *post-hoc* test of the main effect revealed that EMG activity was higher during balancing on either TJ side versus the floor (*p* < 0.001), when all muscle activites were pooled within a condition, but there was no difference between the bladder vs. the flat side of the TJ (*p* = 0.230). The *post hoc* test of the surface by muscle interaction revealed that GM and GastM muscle activities were uniform across the three conditions, and SOL activity was greater in the bladder vs. floor conditon (*p* = 0.009). All other muscles showed greater activity in either TJ condition vs floor, but there were no differences between the bladder and the flat TJ conditons (Figure 2).

### Kinematics

#### Linear acceleration

**FIGURE 3:**
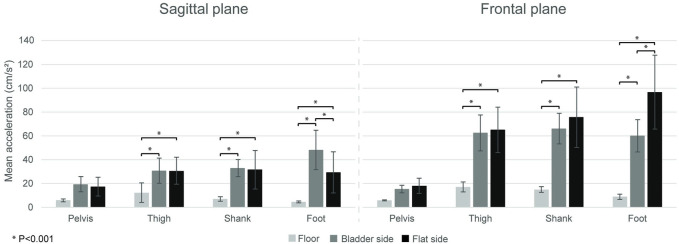
Average linear acceleration (mean ± SD) in the three conditions (floor, and bladder and flat side of the Togu Jumper) for the leg segments (pelvis, thigh, shank, foot) in frontal and sagittal plane. Horizontal bars indicate significant differences (*p* < 0.001).

Results of the statistical analysis declared main effects for surface in frontal plane (*F* = 72.38, *p* < 0.001) and sagittal plane (*F* = 107.47, *p* < 0.001), main effects for segment in frontal plane (*F* = 43.37, *p* < 0.001) and in sagittal plane (*F* = 24.11, *p* < 0.001), furthermore a significant surface by segment interaction both in frontal plane (*F* = 30.17, *p* < 0.001) and in sagittal plane (*F* = 17.64, *p* < 0.001). The *post-hoc* test revealed that in both planes foot acceleration was different among the three conditions (*p* < 0.001): in sagittal plane, the greatest was in the bladder, and the smallest was in the floor condition. In the frontal plane, the greatest was in the flat, and the smallest was in the floor condition. In case of the shank and the thigh segments, in both planes, both TJ conditions revealed higher accelerations, compared to the floor, but there was no difference betweeen the bladder and the flat TJ condtions. No difference was found among the three conditions in the acceleration values of the pelvis in both planes (all *p* > 0.05) (Figure 3).

#### Segmental angular sway

**FIGURE 4:**
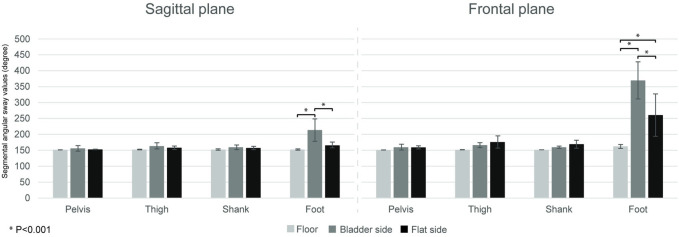
Segmental angular sway over the 10 s stand trials. Bars show the segmental angular sway values (mean ± SD) in the three conditions (floor, and bladder and flat side of the Togu Jumper) for the leg segments (pelvis, thigh, shank, foot) in frontal and sagittal plane. Horizontal bars represented significant differences (*p* < 0.001).

We found significant main effect for surface in both planes (frontal: *F* = 66.92, *p* < 0.001; sagittal: *F* = 20.01, *p* < .001), as well as significant surface by segment interactions (frontal: *F* = 51.54, *p* < 0.001; sagittal plane: *F* = 5.12, *p* = 0.030). In the case of the foot, in frontal plane, the *post-hoc* tests revealed significant differences among the three conditions (*p* < 0.001); in sagittal plane segmental angular sway was higher during balancing on the bladder side vs. the two other conditions (*p* < 0.001) and there was no significant difference between floor and flat side (*p* = 0.260). No difference was found among the three conditions in the case of the shank, thigh, and pelvis in either plane (all *p* > 0.05) (Figure 4).

## Discussion

The main findings of the current study are as follows: i) the use of the TJ instead of the floor induced greater EMG activation in the measured shank and thigh muscles, except in GM, GastM, and SOL, which showed similar activation to the flat condition; ii) the surface condition of the TJ (flat vs. bladder) did not affect the magnitudes of muscle activation; iii) the foot in the sagittal plane showed a greater segmental acceleration in the bladder condition, however in the frontal plane it was higher in the flat condition; iv) and that the segmental angular sway was the highest among all the investigated planes and conditions in the case of the foot segment in the frontal plane in the bladder condition.

Our results demonstrated, that most of the measured shank and thigh muscles showed greater EMG activation while using either TJ side compared to balancing on the floor. The destabilizing effect of standing on an unstable surface is well known. These surfaces are believed to affect the preciseness of somatosensory information from cutaneous mechanoreceptors on the soles of the feet (Wu and Chiang 1997), therefore maintaining equilibrium requires increased activity from the postural muscles, including the muscles in the lower extremities (Loran et al., 2004; Fransson et al., 2007).

We found no significant differences in muscle activations between the two sides of the TJ. In a previous study four different one-legged stance conditions were compared (solid floor, Airex mat, wobble board, and Bosu Balance Trainer bladder side), and the authors found no significant difference in average muscle activation between the Bosu and the wobble board, though they studied merely the peroneal muscle activity (Strom et al., 2016). Our findings are in line with other former studies establishing no differences in ankle muscle activity when balancing on the alternate sides of the Bosu (Laudner and Koschnitzky 2010) or on the Airex pad *versus* uniaxial wobble boards (DeRidder et al., 2015). In contrast, Wolburg and coworkers (2014) have shown that the muscle activity of four lower leg and four upper leg muscles during a one-legged stance differed significantly among five therapy devices with varying stability properties and all muscles required the highest activity for the softest and the most unstable stability trainer, however, they did not investigate wobble board-like devices. On the other hand, the different analysis methodology may also explain the discrapency according to which, in the Wolburg study the mean EMG values for each trial were normalized to the average value of the control condition and expressed as percent control condition and in our research, the average EMG values of the three conditions were presented.

GM activity in our experiment was uniform across the three surface conditions. GM works as a pelvic (Boren et al., 2011) and knee (Sebesi et al., 2021) stabilizer, especially during single-leg standing and unilateral landing on an unstable surface. Its proper function and strength seem to be necessary for maintaining postural stability during one-legged dynamic movements (DiMattia et al., 2005). Despite its importance in stabilization, the use of instability training devices seems to be less effective to improve the strength of the GM, than the hip abductor strength training (Leavey et al., 2010). Few studies have investigated GM activity during a one-legged stance on an unstable surface (Croft et al., 2008; Pfusterschmied et al., 2013) and found that balancing on unstable therapy devices represents no greater demand for this muscle when it is compared to balancing on a less unstable device or solid surface, which is in agreement with our data. In previous studies, it was reported that proximal joints (knee, hip) have an important role under more dynamic and challenging conditions during maintaining a single upright stance on different unstable surfaces (Riemann et al., 2003; Pfusterschmied et al., 2013). However, it was found that significant changes in hip kinematics are accompanied by an enhanced muscle activation only of the biceps femoris, encompassing the hip joint (Pfusterschmied et al., 2013). Our data suggest that even though GM plays a crucial role in a one-legged stance, the use of the TJ does not have significant demand on this muscle, at least, when practitioners perform unilateral standing.

Our results showed no difference in GastM muscle activity while standing on one leg in the three conditions. The three heads of the triceps surae are agonist and, despite that, they differ in many respects. GastM is a superficial and biarticular muscle and its plantar flexor moment depends on the position of the knee as well. It contains a remarkable portion (∼52%) of fast-twitch muscle fibers (Johnson et al., 1973; Buchtal and Schmalbruch, 1980) but the muscle spindle density is lower than in the SOL (Tucker and Turker, 2004; Banks 2006). GastM is intermittently active during balancing and, in this case, its main function is to correct the major interferences of the equilibrium (Ward et al., 2009; Héroux et al., 2014). All above could explain our results and highlight that the three tested conditions did not differ in the disturbance of balance, which we could perceive in the muscle activity of the GastM.

In the current study, SOL activity was higher when balancing on the bladder and soft side of the TJ compared to the solid surface (floor) but there was no difference between the flat TJ side and the floor condition. This is partially in agreement with previous data showing that SOL activity was greater on the compliant balance pad versus on instability devices with a flat, rigid surface on the top side or on the floor (Borreani et al., 2014; Alfuth and Gomoll 2018). In contrast, Cimadoro and coworkers (2013) found higher SOL activity while maintaining a single-leg stance on balance boards with flat, rigid surfaces, when it was compared with the solid floor, but these balance boards had very high instability levels. Unlike the GastM, SOL is a large, deep muscle in the calf (Cohen 2009) and is almost continuously active in standing balance (Joseph et al., 1955). It is composed of mainly (∼88%) slow-twitch muscle fibers (Johnson et al., 1973) which makes it possible to maintain long-term contractions (Power and Howley, 2012). SOL has high muscle spindle density and receives great muscle spindle feedback (Banks 2006), and these are the key to stabilization and postural control, so that triceps surae head is more applied during balancing than the GastM head. Alfuth and Gomoll (2018) suggested, that a compliant device is the most challenging for the SOL when the foot with a plantar flexion immerses into the soft surface and the forefoot becomes unstable. These findings lead to the assumption that the more challenging unstable conditions like the compliant surfaces can provoke greater levels of SOL activity, and the use of the TJ flat side does not seem demanding for the SOL muscle. In terms of muscle activity, there is no difference between the two sides of the TJ, but if the goal is to strengthen the soleus muscle, then balancing on the bladder side is more appropriate.

In the present study, we quantified balance kinematics using the linear acceleration and angular sway of the lower limb segments both in the frontal and sagittal planes during single-leg standing. Other kinematic variables were used previously such as the angular velocity of leg joints (Pfusterschmied et al., 2013), knee joint angle (Alfuth and Gomoll 2018), range of motion variability, peak velocity, direction changes of the ankle (Strom et al., 2016) and center of pressure changes (Cimadoro et al., 2013; Liu et al., 2012; Wolburg et al., 2016; Croft et al., 2008) in unilateral standing conditions with varying stability. In the current study, the linear acceleration data demonstrated movement speed in the medial-lateral leg segment/joint motions in the frontal plane and the anterior-posterior leg segment/joint motions in the sagittal plane. We found that the linear acceleration was greater for both the shank and the thigh when balancing on the TJ (regardless of plane and surface condition) vs. on the floor. This is not surprising, however, the fact that there was no difference in pelvis acceleration among any conditions suggests that despite the rapid movements provoked by the TJ in the lower segments has no effect on pelvis acceleration. This, on one hand, is supported by our EMG results showing no differences in mean GM muscle activity among the three surface conditions. On the other hand, others have shown, that during a one-legged stance on rigid or on unstable surfaces, the control of the center of pressure was more prevalent at the ankle than at the hip or knee and the latter two seem to play an important role under more dynamic and challenging conditions (Riemann et al., 2003; Croft et al., 2008), e.g., balancing on an unstable therapy device without a fixed base of support (Pfusterschmied et al., 2013). Therefore, our and previous findings suggest that neither side of the TJ count for a very challenging unstable condition of the knee and hip, if compared to the floor during a one-leg stance, therefore, for pelvic proprioceptive training a device with a higher instability level or more dynamic balance exercises would be more effective.

We found that when balancing on the bladder side of the TJ, foot acceleration was higher in the sagittal plane (flexion-extension), however, on the flat side we received greater acceleration values in the frontal plane (eversion-inversion). A possible explanation for the different kinematics could be that a compliant surface lets immerse the foot into the bladder side and stretches the plantar fascia of the sole, unlike the other side, where the fascia maintains its normal stiffness. The plantar fascia has an important role in sensorimotor regulation of postural control in standing (Erdemir et al., 2004; Stecco et al., 2013) and a temporary increase in fascial stiffness could enhance fascial proprioception and muscle activity (Schleip et al., 2005). This is in line with our EMG results, according to which, on the bladder side of the TJ, we found higher SOL activity and this could support the quicker flexion-extension motions. On the flat side, in the absence of stretched plantar fascia and enhanced SOL activity, the sudden eversion-inversion motions may help to maintain equilibrium. Further research is needed to clarify how a compliant surface influences the balancing mechanisms.

We recorded the segmental angular sway in time to quantify the magnitudes of segmental movements around joints. Both in the frontal and sagittal planes the surface did not affect the segmental angular sway of the shank, thigh, and pelvis. Most of the studies examine joint angles, direction changes, range of motion, etc. (Blackburn et al., 2003; Pfusterschmied et al., 2013; Alfuth and Gomoll 2018), and to the best of our knowledge, no previous studies have been reported on the segmental angular sway while balancing on one leg on the TJ. Fransson and coworkers (2007) showed that task difficulty affected joint movement more in the knee than in hip. Knee joint kinematics can differ when balancing on equipment with different stability characteristics (Pfusterschmied et al., 2013). The corrective action of the knee joint becomes very important when one-leg balancing became more challenging (Riemann et al., 2003). The results above suggest that there was not enough difference among the three investigated conditions that would have resulted in a different amount of movements in the shank, thigh, and pelvis segments.

In the case of the foot segment, in the frontal plane, our results showed differences in the segmental angular sway among each of the three conditions. In the sagittal plane, the segmental angular sway was the highest on the bladder side compared to the other two conditions, and we found no difference between the flat side of the TJ and the floor. The current results show that the foot is the most motile leg segment during a one-legged barefoot stance on the floor or on unstable devices, and the quantity of the foot motions were the highest on the bladder side of the TJ in both investigated planes. This is supported by previous data showing that equilibrium is essentially controlled by movements of the ankle with minimal participation of the hip or knee joint (Williams et al., 2001; Riemann et al., 2003). Our results highlighted that these balancing ankle motions derive principally from the movements of the foot and not from the shank, furthermore the bladder side of the TJ has a higher difficulty level compared to the flat side in terms of the quantity of the foot motions.

## Limitations

An important limitation in the present study is that we used linear accelerations to express how rapidly the segments moved in a specific plane during balancing. Though we quantified segmental angular sway to express rotational movement in the segments, we did not provide data on angular accelerations, i.e., how fast joint rotations were developed. Another limitation is that we studied only healthy recreationally trained females and that the present data cannot be generalized to populations such as males, athletes, or injured individuals.

## Conclusion and Perspectives

In summary, the present study shows that balancing on the two sides of the TJ has no effect either on the kinematics of the pelvic segment or on the EMG activity of the GM if it is compared to the floor, therefore more challenging devices or other exercise postures seem to be required for the proprioceptive training of this region. The use of the bladder and the flat side of the TJ is similarly appropriate to train thigh muscles (VM, VL, BF) and the invertor and evertor muscles of the lower leg (TA, PL). When the goal of the therapy or training is the specific re-establishment of the neuromuscular deficiency of the SOL, the application of the TJ bladder side might be more effective. In terms of kinematics, the balance strategies during one leg standing on the two sides of the TJ differed only in the case of the foot. In the frontal plane, the foot moves faster on the flat side of the device, so after injuries of the ankle ligaments, it is recommended to use the bladder side of the TJ first, as we balance on it with slower, more controlled movements. Later, the flat side can be used to make balancing tasks more difficult. Using alternately both sides of the TJ is beneficial for improving balance, rehabilitation, or prevention of lower extremity injury because they can complement each other’s effects. Further research is required to investigate the angular accelerations, the influence of health and training status as well as gender on balance strategy during one-legged stance on the TJ sides.

## Data Availability

The raw data supporting the conclusion of this article will be made available by the authors, without undue reservation.

## Abbreviations

BBT, BOSU balance trainer; BF, Biceps femoris muscle; EMG, Electromyography; GastM, Gastrocnemius medialis muscle; GM, Gluteus medius muscle; PL, Peroneus longus muscle; SOL, Soleus muscle; TA, Tibialis anterior muscle; TJ, Togu Jumper; VL, Vastus lateralis muscle; VM, Vastus medialis muscle.
